# Draft genome sequence of *Mammaliicoccus sciuri* strain 30RR isolated from mastitic buffalo milk

**DOI:** 10.1128/mra.00361-26

**Published:** 2026-07-24

**Authors:** Md. Mahfujur Rahman, Md. Shahidur Rahman Chowdhury, Hemayet Hossain, Sabbir Khan, M. Nazmul Hoque

**Affiliations:** 1 Department of Medicine, Sylhet Agricultural University 399806, Sylhet, Bangladesh; 2 Department of Veterinary Science and Animal Husbandry, Teesta University, Rangpur, Bangladesh; 3 Molecular Biology and Bioinformatics Laboratory, Department of Gynecology, Obstetrics and Reproductive Health, Gazipur Agricultural University 198780, Gazipur, Bangladesh; University of Manitoba, Winnipeg, Manitoba, Canada

**Keywords:** *Mammaliicoccus sciuri*, buffalo milk, biosynthetic gene clusters, novel sequence type

## Abstract

We report the draft genome sequence of *Mammaliicoccus sciuri* strain 30RR, isolated from the milk of a buffalo with clinical mastitis. The ~2.5 Mbp genome of strain 30RR was assembled into 40 contigs and comprises approximately 2,600 predicted genes, including two antimicrobial resistance genes and four biosynthetic gene clusters.

## ANNOUNCEMENT

Recently reclassified from *Staphylococcus* through core genome analysis, *Mammaliicoccus sciuri* is a gram-positive opportunistic pathogen of clinical and agricultural concern. Although historically regarded as a benign commensal, it is now recognized as an opportunistic pathogen capable of causing mild-to-severe infections in both human and animal hosts (1–3). In dairy settings, *mecA*-harboring strains now dominate bovine milk samples, often carrying unique non-typeable staphylococcal cassette chromosome mec (SCCmec) elements mediating β-lactam resistance (3, 4). Despite genomic reports from Europe and Southeast Asia, South Asian data are limited. We present the draft genome of *M. sciuri* 30RR, isolated from mastitic buffalo milk in Sylhet, Bangladesh (24.54°N, 91.52°E) during a surveillance study conducted from June 2023 to May 2024.

Approximately 10 mL of milk was aseptically collected from a buffalo cow with clinical mastitis, as examined by the California Mastitis Test (5, 6). Subsequently, 100 µL of the sample was enriched in 5 mL of nutrient broth and incubated overnight at 37°C under aerobic conditions (6–8). After enrichment, 2 µL of broth was streaked onto nutrient agar (HiMedia, India) and incubated at 37°C for 24 h. The enriched broth was serially diluted up to 10^−^³ and streaked onto mannitol salt agar plates (Oxoid, UK) for overnight incubation at 37°C (8, 9). Colonies displaying typical staphylococcal morphology (creamy-white to yellow, convex, and opaque) were selected and purified by repeated subculture (8). Presumptive genus assignment relied on Gram staining (gram-positive cocci in clusters) and biochemical testing, *viz*. catalase (positive), coagulase (negative), and mannitol fermentation (positive), consistent with *Mammaliicoccus* (4, 8). Species-level identification was subsequently performed using the VITEK-2 system v9.01 (bioMérieux, France), which employs automated biochemical profiling of a 0.5 McFarland standardized bacterial suspension using species-specific identification cards. Finally, definitive species confirmation was provided by whole-genome sequencing. Genomic DNA was extracted from a pure overnight culture using the QIAamp DNA Mini Kit (Qiagen, Germany) following the manufacturer’s protocol. DNA purity and concentration were assessed by NanoDrop 2000 (Thermo Fisher Scientific, USA) (4). Libraries (1 ng DNA; Nextera DNA Flex Kit, Illumina) were sequenced on the Illumina MiSeq platform (2 × 150 bp), generating 996,952 read pairs (2,897,300 bp). Reads were quality-assessed with FastQC v0.12.1 (10) and trimmed with Trimmomatic v0.40 (LEADING:20, TRAILING:20, SLIDINGWINDOW:4:20, and MINLEN:50) (11). Genome assembly was performed with SPAdes v4.2.0 (12) and annotated using the NCBI Prokaryotic Genome Annotation Pipeline v.6.10 (13). Genome completeness, antimicrobial resistance genes (ARGs), virulence factor genes, plasmids, pathogenicity, CRISPR arrays, and metabolic features were analyzed using CheckM v.1.2.4 (14), CARD v4.0.1 (15), VFDB v6.0 (16), PlasmidFinder v2.0.1 (17), PathogenFinder2 v0.5.0 (18), CRISPRCasFinder v2.0.3 (19), and RAST v2.0 server (20), respectively, with default parameters unless otherwise stated. multi-locus sequence typing (MLST) was performed using mlst (https://github.com/tseemann/mlst) with the *M. sciuri* (msciuri) seven-locus scheme. SCCmec elements were typed with SCCmecFinder (21). Biosynthetic gene clusters were predicted with antiSMASH v8.0 (22). The genomic features of *M. sciuri* 30RR are summarized in Table 1. MLST revealed a putative novel sequence type with three novel alleles (*glpK*-120, *gmk*-61, and *pta1*-129), and antiSMASH predicted four biosynthetic gene clusters. PathogenFinder2 classified the strain as non-pathogenic (score, 0.439).

**TABLE 1:** Genomic features of *Mammaliicoccus sciuri* strain 30RR isolated from buffalo milk

Feature	Value
GenBank accession no.	JBWGSY000000000
BioProject accession no.	PRJNA1434438
BioSample accession no.	SAMN56400945
SRA accession no.	SRR37536882
Sequencing platform	Illumina MiSeq
No. of raw read pairs	996,952
No. of trimmed read pairs	976,858 (97.98% retention)
Sequencing output (bp)	2,897,300
Assembled genome size (bp)	2,545,126
GC content (%)	32.35
No. of contigs (≥500 bp)	40
N_50_ (bp)	116,238
L_50_	6
Mean coverage (×)	99.66
BUSCO completeness (%)	100
Total annotated features	2,600
Coding sequences (CDS)	2,565
Ribosomal RNAs (rRNA)	4
Transfer RNAs (tRNA)	30
Transfer-messenger RNA (tmRNA)	1
ARGs	*mecA1* and *sal(A*)
Virulence factor genes	None detected
Biosynthetic gene clusters	4 (NI-siderophore, cyclic-lactone autoinducer, two terpene)
CRISPR-Cas system	Partial Type II-U (five low-evidence arrays; 1 Cas9 gene, contig_13)
PathogenFinder2 score	0.439 ± 0.138 (non-pathogen)
MLST scheme (msciuri)	ack-30, aroE-55, ftsZ-2, glpK-120^*a*^, gmk-61^*a*^, pta1-129^*a*^, and tpiA-27

^*a*^ Novel alleles deposited in PubMLST (submissions BIGSdb 20260312192623, 20260312190228, and 20260312191502).

## Data Availability

This whole genome shotgun project has been deposited in GenBank under the accession no. JBWGSY000000000. The version described in this paper is the first version, JBWGSY010000000.1. Raw sequencing reads are available under SRA accession no. SRR37536882. The BioProject and BioSample accession numbers are PRJNA1434438 and SAMN56400945, respectively.
